# Improved Specificity and Safety of Anti-Hepatitis B Virus TALENs Using Obligate Heterodimeric *Fok*I Nuclease Domains

**DOI:** 10.3390/v13071344

**Published:** 2021-07-12

**Authors:** Tiffany Smith, Prashika Singh, Kay Ole Chmielewski, Kristie Bloom, Toni Cathomen, Patrick Arbuthnot, Abdullah Ely

**Affiliations:** 1 Wits/SAMRC Antiviral Gene Therapy Research Unit, School of Pathology, Faculty of Health Sciences, University of the Witwatersrand, Parktown 2193, South Africa; tiffsmith7777@gmail.com (T.S.); prashika.singh.ps@gmail.com (P.S.); kristie.Bloom@wits.ac.za (K.B.); patrick.arbuthnot@wits.ac.za (P.A.); 2 Institute for Transfusion Medicine and Gene Therapy, Medical Center-University of Freiburg & Medical Faculty, University of Freiburg, 79106 Freiburg, Germany; kay.chmielewski@uniklinik-freiburg.de (K.O.C.); toni.cathomen@uniklinik-freiburg.de (T.C.)

**Keywords:** HBV, cccDNA, obligate heterodimeric TALENs, Sharkey

## Abstract

Persistent hepatitis B virus (HBV) infection remains a serious medical problem worldwide, with an estimated global burden of 257 million carriers. Prophylactic and therapeutic interventions, in the form of a vaccine, immunomodulators, and nucleotide and nucleoside analogs, are available. Vaccination, however, offers no therapeutic benefit to chronic sufferers and has had a limited impact on infection rates. Although immunomodulators and nucleotide and nucleoside analogs have been licensed for treatment of chronic HBV, cure rates remain low. Transcription activator-like effector nucleases (TALENs) designed to bind and cleave viral DNA offer a novel therapeutic approach. Importantly, TALENs can target covalently closed circular DNA (cccDNA) directly with the potential of permanently disabling this important viral replicative intermediate. Potential off-target cleavage by engineered nucleases leading to toxicity presents a limitation of this technology. To address this, in the context of HBV gene therapy, existing TALENs targeting the viral *core* and *surface* open reading frames were modified with second- and third-generation *Fok*I nuclease domains. As obligate heterodimers these TALENs prevent target cleavage as a result of *Fok*I homodimerization. Second-generation obligate heterodimeric TALENs were as effective at silencing viral gene expression as first-generation counterparts and demonstrated an improved specificity in a mouse model of HBV replication.

## 1. Introduction

Site-specific cleavage of DNA with engineered nucleases forms the basis of gene editing techniques that are being developed to inactivate replication of hepatitis B virus (HBV) [1]. Chronic infection with HBV is an important global health problem, and currently available therapies have modest curative efficacy [2]. Fatal complicating cirrhosis and hepatocellular carcinoma are common amongst carriers of the virus, and account for approximately 887,000 HBV-related annual global deaths. Persistence of the essential HBV replication intermediate comprising covalently closed circular DNA (cccDNA) and minimal effects of licensed antivirals on this intermediate are the main reasons for current difficulties with eliminating HBV infection. Designer nucleases used to target cccDNA include zinc finger nucleases (ZFNs), transcription activator-like effector nucleases (TALENs), and clustered regularly interspaced short palindromic repeats (CRISPR) with CRISPR-associated (Cas) proteins [1]. Targeted mutation is typically initiated by cleavage of a specific DNA sequence, which is then repaired by non-homologous end joining (NHEJ). With repeated cutting, error-prone DNA repair eventually leads to irreversible formation of replication-disabling insertions and deletions (indels) at the cleavage site.

CRISPR/Cas is now the most commonly applied gene editing tool, and the ease with which targeting nucleases may be generated is an important reason for popularity of the technology. These RNA-guided endonucleases have successfully been used to target DNA of HBV, and evidence indicates that cccDNA may be disabled in cells replicating the virus (reviewed in [3]). However, a potential complication for therapy is the pervasive pre-existing immunity to the endonucleases derived from commensal *Streptococcus pyogenes* or *Staphylococcus aureus* [4,5]. Consequently, in vivo efficacy of candidate therapeutic gene editors may be compromised following systemic administration of sequences encoding anti-HBV CRISPR/Cas. Because TALENs and ZFNs are proteins derived from plant-infecting *Xanthomonas* species or naturally occurring zinc finger proteins, pre-existing immunity is likely to be uncommon. To avoid immune attenuation, use of these gene editors may therefore be preferable to disable HBV. Comparisons between ZFNs and TALENs show that TALENs have advantages over ZFNs: unlike with ZFNs, DNA binding by individual TALEN monomers is not influenced by neighboring sequences, and TALENs have better specificity for their cognates than ZFNs [6,7].

Although gene editing technology has impressive potential, ensuring specificity of action is vital for therapeutic application. Off-target mutation caused by imprecise cleavage needs to be minimized to prevent potentially serious unintended consequences. Various approaches have been employed to improve precision of designer nucleases. In the case of CRISPR/Cas shortening of the guide sequence [8], combining Cas nickases with two guides [9,10], inclusion of a hairpin structure in guide sequences [11], and the recently described prime editing approach [12] have all been used to achieve this goal. In the case of ZFNs and TALENs, the *Fok*I catalytic domain has been engineered in various ways to improve specificity. Slowing kinetics of target cleavage by *Fok*I, thereby selectively reducing action at low affinity off-target binding sites of ZFNs, has been successfully employed [13]. Shortening the duration of action of gene editors may also diminish off-target effects, and this may be achieved by using mRNA as the coding nucleic acid [14]. Modifying the *Fok*I nuclease domains to ensure formation of obligate heterodimers has also been utilized [15,16,17]. The rationale for this approach is that juxtaposition of duplex-cleaving homodimers, comprising two left or two right subunits at an off-target site, is prevented. To avert generation of homodimers, researchers modified amino acid sequences at the interface between nuclease domains of these ZFNs, such that duplex-cleaving *Fok*I subunits were only active when heterodimers were assembled [15,16,17]. In a similar vein, directed evolution has been employed to improve catalytic activity of the *Fok*I nuclease domain and yielded so-called Sharkey nuclease domains [18]. We employed these approaches to improve specificity and activity of TALENs acting against HBV DNA by generating gene editors that require formation of obligate TALEN heterodimers to be active on their viral cognates. Evaluation in cultured cells and in vivo showed that the modified TALENs had similar activity to the first-generation counterparts, but with improved specificity to targets.

## 2. Materials and Methods

### 2.1. Plasmids

pCH-9/3091 [19] is a replication-competent plasmid containing a greater-than-genome length HBV sequence. Transcription of the pCH-9/3091 plasmid is driven from the CMV promoter and yields a greater-than-genome length transcript that resembles the viral pgRNA, which subsequently initiates viral replication. pCI-neo eGFP [20] and pCI-neo FLuc [21] have been described before. Second-generation obligate heterodimeric TALENs and third-generation obligate heterodimeric TALENs with Sharkey mutations were derived from existing first-generation anti-HBV TALENs targeted against the *core* and *surface* ORFs of the viral genome [22]. Each first-generation TALEN consists of left and right monomers comprising a DNA-binding TALE array fused to a first-generation *Fok*I nuclease domain with a hemagglutinin (HA) epitope and a nuclear localization signal (NLS) located at the N-terminus [22]. The left and right monomers of the *core*-targeting TALEN bind to nucleotides 2319–2337 and 2351–2369 of the HBV genome. The left and right monomers of the *surface*-targeting TALEN bind to nucleotides 411–429 and 443–452 of the HBV genome [22]. The first-generation anti-HBV TALENs exist within the pVAX plasmid backbone [23] and are expressed from the CMV promoter. Second- and third-generation TALENs were generated by substituting the first-generation *Fok*I nuclease-encoding sequence in the pVAX plasmids with the second-generation obligate heterodimeric *Fok*I nuclease domain sequences or third-generation obligate heterodimeric *Fok*I nuclease domain sequence with Sharkey mutations, respectively.

### 2.2. Cell Culture

Huh7 cells were cultured in low-glucose DMEM (Thermo Scientific, Waltham, MA, USA), and HEK293 and HepG2.2.15 cells were maintained in high-glucose DMEM (Thermo Scientific, CA, USA). Growth medium was supplemented with penicillin (100,000 U/mL), streptomycin (100 μg/mL), and 10% Gibo™ FBS (Thermo Scientific, Waltham, MA, USA). Cells were maintained at 37 °C and 5% CO_2_ in a humidified incubator.

### 2.3. Immunofluorescence Detection of Anti-HBV TALEN Expression

Twenty-four hours before transfection, Huh7 cells were seeded in a 96-well plate at a density of 50% per well. Lipofectamine^®^ 3000 (Thermo Scientific, Waltham, MA, USA) was used to transfect 100 ng of each TALEN monomer-expressing plasmid. pCI-neo eGFP was transfected separately to verify success of transfection. Forty-eight hours after transfection, TALEN expression was assessed by immunofluorescence detection of the HA epitope using an anti-HA primary antibody (Sigma-Aldrich, St. Louis, MO, USA) and Alexa Fluor 448-labeled secondary antibody (Thermo Scientific, Waltham, MA, USA). Fluorescence was detected using the Axiovert 100M fluorescence microscope (Zeiss, Oberkochen, Germany).

### 2.4. Assessment of HBV Silencing in Cultured Cells by ELISA

Huh7 cells were seeded in a 6-well plate at a cell density of 50% and transfected one day later. Using Lipofectamine^®^ 3000, cells were transfected with 1 µg of each left TALEN monomer-expressing plasmid together with 1 µg of its cognate right TALEN monomer-expressing plasmid, 300 ng of pCH-9/3091, and 200 ng of pCI-neo eGFP. As a mock, 2 µg of pUC118 (Addgene, Watertown, MA, USA) was co-transfected with pCH-9/3091 and pCI-neo eGFP. Forty-eight hours post-transfection, successful transfection was determined by visualizing GFP expression and HBsAg secretion. GFP expression was detected using fluorescence microscopy and HBsAg was assayed using ELISA with the Monolisa™ HBsAg ULTRA kit (Bio-Rad, Hercules, CA, USA).

### 2.5. On-Target Cleavage by Anti-HBV TALENs Using the SURVEYOR Assay

HepG2.2.15 cells were seeded in 6-well plates and transfected the following day with 200 ng pCI-neo eGFP and 1 µg of each of the left and right TALEN monomer-encoding plasmids using Lipofectamine^®^ 3000. As a control, 2 µg of pUC118 was transfected in place of the TALEN-expressing plasmids. Spent medium was replaced three days post-transfection. After five days, half the cells were harvested and the other half re-seeded. Transfection and re-seeding were repeated for an additional 2 cycles. After the final transfection, supernatants were collected for HBsAg ELISA, and cells were harvested and used to assess targeted cleavage. Total DNA was extracted from HepG2.2.15 cells as previously described [22]. Sequences comprising 520 bp and spanning ≈260 bp upstream and ≈260 bp downstream of the predicted target sites for the C and S TALENs were amplified under standard PCR conditions. The following primer sets were used: Core forward 5′-GAA CTA ATG ACT CTA GCT ACC T-3′, Core reverse 5′-CCT ACA AAC TGT TCA CAT TT-3′; Surface forward 5′-CCT AGG ACC CCT TTC TCG TGT-3′, and Surface reverse 5′-ACT GAG CCA GGA GAA ACG GG-3′. Three hundred nanograms of PCR products were subjected to heteroduplex formation by denaturation at 95 °C followed by cooling to 35 °C at a ramp rate of −0.1 °C/s, then holding at 35 °C for 2 min. At this point, PCR products were incubated on ice for 5 min, followed by the addition of 1 µL of CelI enzyme and 2 µL of 10× NEB buffer 2 (New England Biolabs, Ipswich, MA, USA). Samples were held at 4 °C for 10 min, followed by heating and incubation at 37 °C for 25 min. Cleaved products were resolved using agarose gel electrophoresis, and ImageJ software was used to measure targeted disruption as previously described [24]. As a positive control for the Surveyor assay, heteroduplexes were formed by the PCR amplification of first-generation and mutant HBx sequences [25]. First-generation and mutant amplicons were mixed at equimolar amounts and denatured and annealed to form heteroduplexes.

### 2.6. Assessment of Cell Viability by MTT Assay

HEK293 cells were seeded in a 96-well plate at a density of 30% six hours prior to transfection. Polyethylenimine (PEI) (0.1 mg/mL) was used to co-transfect 15 ng of pCH-9/3091, 15 ng of pCI-neo eGFP, and 85 ng of the left and right TALEN monomer-expressing plasmids. Cells transfected with 170 ng of pUC118 served as the mock transfection control. Cells treated with 50% dimethyl sulfoxide (DMSO) were used as a positive control, and untreated cells served as the negative control. Cell viability was assessed 48 h after transfection. Twenty microliters of 5 mg/mL MTT, made up in PBS, was added to each well and incubated at 37 °C for 1 h. Culture medium was subsequently removed, 200 µL of DMSO added and the cells incubated for a further 5 min. The metabolism of MTT to form blue formazan was determined by measuring the optical densities at 570 nm and 655 nm using an iMARK™ Microplate reader (Bio-Rad, Hercules, CA, USA).

### 2.7. Animal Studies

Anti-HBV TALEN efficacy was assessed using the murine hydrodynamic injection (HDI) model of HBV replication. All experiments on animals were conducted in accordance with protocols approved by the University of the Witwatersrand Animal Research Ethics Committee. HDI was performed on 5-week-old female NMRI mice, weighing between 20 and 30 g. The injectate comprised a plasmid DNA-containing saline solution equal to 10% of the body weight of each mouse (final volume of 2–3 mL). The DNA/saline solutions contained 5 µg HBV target DNA (pCH-9/3091), 5 µg pCI-neo FLuc, and either 20 µg of pUC118 or 10 µg of each corresponding left and right TALEN-expressing plasmids. All plasmids were prepared using the Endo-Free Plasmid Maxi kit (Qiagen, Hilden, Germany). To confirm hepatic delivery of the plasmids, 3 days post-injection mice were injected intraperitoneally with 150 mg/kg of D-luciferin (PerkinElmer, Inc., Waltham, MA, USA) and bioluminescence imaging carried out using an IVIS Kinetic In Vivo Optical Imaging System (PerkinElmer, Inc., Waltham, MA, USA). Blood was collected from mice by retro-orbital puncture on days 3 and 5 post-injection, and the serum was then diluted in an equal volume of saline. One hundred microliters were used to measure serum HBsAg concentrations using the Monolisa™ HBsAg ULTRA kit. Serum ALT levels were quantified using a kinetic assay with an automated photometric analyzer (Roche Applied Science, Penzberg, Germany). Mice were euthanized on day 5 by CO_2_ exposure, and livers were then immediately harvested. To assess targeted cleavage in murine samples, we dissected and mechanically homogenized 25 mg of liver. Total DNA was extracted from liver homogenates using the QIAamp^®^ DNA Blood Mini Kit (Qiagen, Hilden, Germany). On-target cleavage was assessed using the Surveyor assay as described earlier.

### 2.8. Quantification of Circulating VPEs and Gene Expression

To quantify viral particle equivalents (VPEs), we extracted total DNA from 50 µL of diluted serum using the QIAamp^®^ DNA Mini Kit. Circulating VPEs from experimental and control mice were measured by qPCR using the CFX96 Touch™ Real-Time PCR Detection System (Bio-Rad, Hercules, CA, USA). The Acrometrix HBV Panel (Thermo Scientific, Waltham, WA, USA) was used as a standard for quantitation. DNA samples were subjected to real-time PCR using 2× FastStart Essential DNA Green Master (Roche Applied Science, Penzberg, Germany) with the following primers: HBVs F 5′-TGC ACC TGT ATT CCC ATC-3′ and HBVs R 5′-CTG AAA GCC AAA CAG TGG-3′. To assess intrahepatic HBV gene expression, we quantified viral RNA levels by RT-qPCR. Total cellular RNA was extracted from liver homogenates using the TRIzol^®^ Reagent (Thermo Scientific, Waltham, WA, USA) and reverse transcribed using the QuantiTect reverse transcription kit (Qiagen, Hilden, Germany). The cDNA samples were subjected to real-time PCR using 2× FastStart Essential DNA Green Master. Viral surface and pregenomic RNA were amplified using the HBVs F and R primers, and a second primer set (BCP F 5-′ACC ACC AAA TGC CCC TAT-3′ and BCP R 5′-TTC TGC GAG GCG GCG A-3′) was used to amplify pregenomic RNA selectively. Murine *GAPDH* was amplified with the mGAPDH F 5′-TTC ACC ACC ATG GAG AAG GC-3′ and mGAPDH R 5′-GGC ATG GAC TGT GGT CAT GA-3′ primers to relativize viral RNA levels.

### 2.9. Assessment of on- and off-Target Mutagenesis by Next Generation Sequencing

Potential off-target sites within the host genome were predicted for the S and C TALENs using PROGNOS software [26], and the top four off-target sites were selected for further analysis. Four mice were chosen from each group and total DNA was extracted from homogenized liver samples using the QIAamp^®^ DNA Blood Mini Kit. Sequences flanking the on-target site and potential off-target sites were amplified using the KAPA HiFi HotStart ReadyMix (Kapa Biosystems, Wilmington, MA, USA) with primer sets listed in Table S1. The primers were designed to amplify a 250–300 bp region flanking the on-target site and each of the four off-target sites. The PCR amplicons were column-purified using the MinElute Gel Extraction kit and pooled on the basis of group and target amplicon. Primer sets contained Multiplex IDentifiers (MIDs) to allow discrimination of the different mice from each other. Each amplicon had a 10 bp MID flanking the different regions of interest. Samples were sequenced using the HiSeq 2500 System (Illumina, San Diego, CA, USA). The generated paired-end reads were merged using Flash (https://ccb.jhu.edu/software/FLASH/; October 2020 to June 2021) and demultiplexed according to the MIDs that were used for each sample (https://github.com/najoshi/sabre, accessed on 30 June 2021). Merged reads were further analyzed using the command line version of CRISPResso2 [27] with a window size of 30, substitutions were ignored.

### 2.10. Data Analysis

Data were presented as mean ± SEM. Two-tailed Student’s *t*-tests were performed using GraphPad Prism 4.0 (GraphPad Software Inc., San Diego, CA, USA) for the comparison between two groups. A value of *p* < 0.05 (*) was considered statistically significant.

## 3. Results

### 3.1. Targeted Inhibition of HBV S Expression in Cultured Mammalian Cells

**Figure 1:**
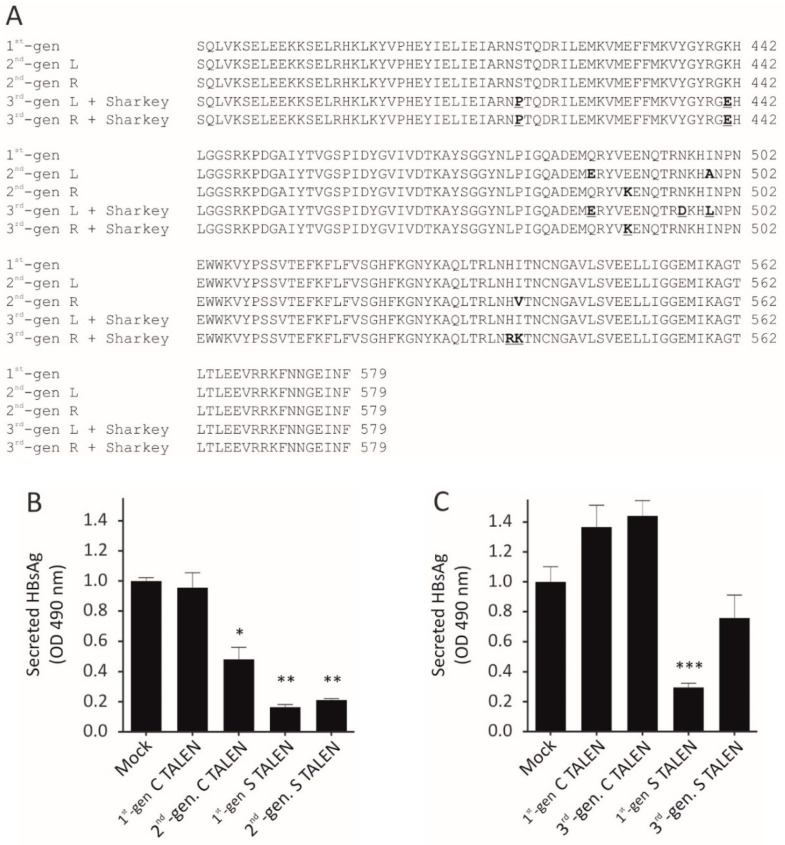
(**A**) Multiple sequence alignment of first-, second-, and third-generation *Fok*I nuclease domains showing obligate heterodimeric (bold) and Sharkey (bold and underlined) modifications. (**B**) To compare silencing efficacy of second-generation TALENs, Huh7 cells were transiently co-transfected with the pCH-9/3091 and the indicated plasmids. HBsAg was assayed 48 h after transfection. Data obtained were averaged, and the means were normalized to the mock. Statistical Scheme 0. * = p < 0.05; ** = *p* < 0.001; *** = *p* < 0.001. (**C**) As in (**B**), but comparing third-generation against anti-HBV TALENs containing the first-generation *Fok*I nuclease domain.

Second-generation TALENs were successfully generated by substituting the first-generation *Fok*I nuclease domains with obligate heterodimeric *Fok*I nuclease domains. Left TALEN monomers contained the Q486E and I499A mutations, whereas right monomers contained the E490K and I538V mutations [16], ensuring that these *Fok*I nuclease domains are only functional as left/right heterodimers (Figure 1a and Figure S1). Third-generation TALENs contained the KKR and ELD obligate heterodimeric residues [17], as well as the Sharkey mutations (S418P and K441E) (Figure 1a). The second- and third-generation *Fok*I nuclease domains within the anti-HBV TALENs were assessed in Huh7 cells after transfection. Immunofluorescence detection of the HA epitope confirmed that the TALENs were expressed in cell culture (Figure S2a). Secreted HBsAg was significantly reduced in culture supernatants of cells transfected with first-, second-, and third-generation *surface*-targeting TALENs (Figure 1b,c). The second-generation obligate heterodimeric TALENs exhibited silencing equivalent to that of the first-generation TALEN. Although inhibition of HBV gene expression by the Sharkey S TALEN was significant, efficacy was lower than that observed for the first-generation S TALEN. As expected, first-generation and Sharkey TALENs targeted against the *core* ORF did not affect suppression of HBsAg secretion. In contrast, the second-generation obligate heterodimeric C TALEN reduced HBsAg levels by 50%. Since the C TALENs are targeted to the *core* ORF, the observed suppression cannot be as a result of disruption of the *surface* ORF and likely reflects a transcriptional inhibitory mechanism. Inhibition of HBsAg secretion was independent of cellular toxicity as measured by cell viability assays (Figure S2b).

**Figure 2:**
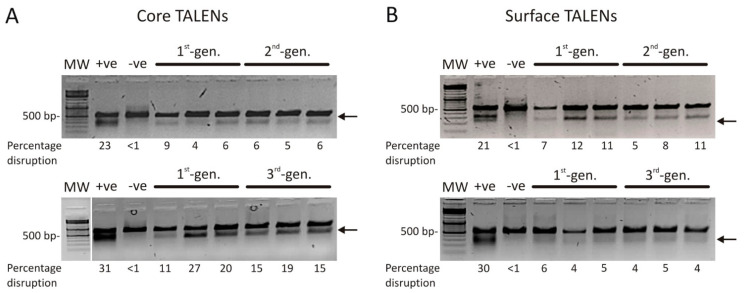
Targeted cleavage of viral DNA in cultured cells. Analysis of targeted mutation sequences targeted by second- (**A**) and third- (**B**) generation TALENs employing the Surveyor assay. Arrows depict cleaved heteroduplexes. MW: molecular weight marker, +ve: HBx heteroduplexes, −ve: mock transfection.

To assess targeted disruption by the second- and third-generation *Fok*I nuclease-domain containing TALENs, we used the Surveyor assay to quantify indels at the *core* and *surface* target sites. The Surveyor assay employs the CelI enzyme from celery to cleave heteroduplex DNA and thereby quantitatively measures indel formation. As Huh7 cells transfected with pCH-9/3091 do not yield sufficient HBV DNA for accurate quantitation, the HepG2.2.15 cell line was used for these experiments. HepG2.2.15 cells, which have a stably integrated greater-than-genome-length HBV sequence and constitutively model viral replication, were transfected with first-, second-, and third-generation TALENs sequentially in triplicate. C and S TALEN target sites were amplified by PCR from extracted DNA and treated with CelI to cleave heteroduplex DNA, which was used to calculate percentage indels (Figure 2). All TALENs exhibited site-specific targeted mutagenesis, as evidenced by cleavage of heteroduplexes, and head-to-head comparisons indicated there was no statistically significant difference in cleavage efficacy when using second- or third- generation anti-HBV TALENs (2nd-gen C vs. 1st-gen C: *p* = 0.6779; 3rd-gen C vs. 1st-gen C: *p* = 0.4929; 2nd-gen S vs. 1st-gen S: *p* = 0.2254; 3rd-gen S vs. 1st-gen S: *p* = 0.5286). The results from the Surveyor assay confirm that second- and third-generation TALENs function as designed and cause targeted disruption at intended sites within HBV DNA. As a result of the highly active and constitutive nature of viral replication in this model, suppression of HBsAg secretion is difficult to achieve (Figure S3).

### 3.2. TALEN-Mediated HBV Silencing in Mice

**Figure 3:**
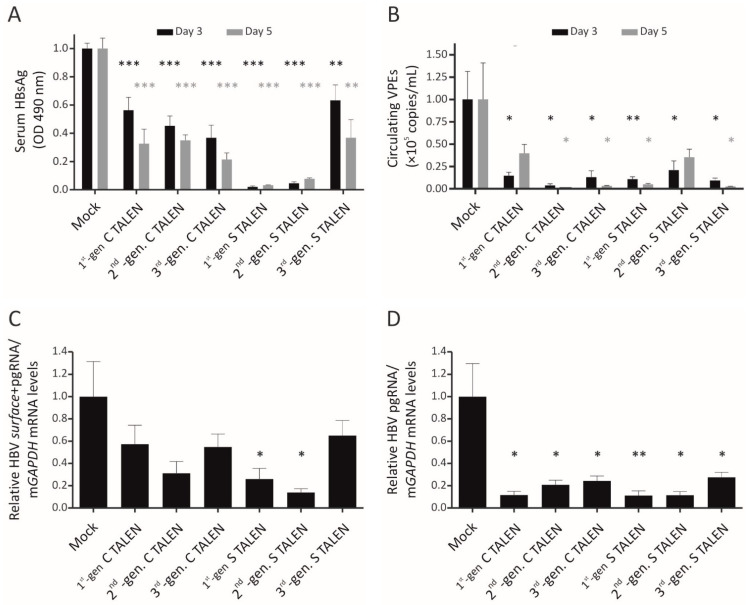
Inhibition of markers of HBV replication in vivo. NMRI mice were hydrodynamically co-injected with pCH-9/3091 and pUC118 or TALEN-encoding plasmids. (**A**) Blood samples were collected on day 3 and day 5 post-injection, and HBsAg levels in the serum were assessed using ELISA. (**B**) Total DNA was extracted from the serum of mice, and qPCR was used to determine the circulating viral particle equivalents (VPEs). (**C**,**D**) RNA was extracted from murine livers on day 5 post-HDI, and HBV mRNA concentrations were assessed by qPCR. Primers designed against the *S* and *C* ORFs were used to measure (**C**) pgRNA and *surface* mRNA levels or (**D**) pgRNA levels only and relativized to m*GAPDH* mRNA. Means were calculated and normalized to the mock. n = 7 (mock, 1st-gen S and 3rd-gen S), n = 6 (2nd-gen S and 3rd-gen C), or n = 5 (1st-gen C and 2nd-gen C). Statistical significance was calculated in comparison to the mock (* = *p* < 0.05; ** = *p* < 0.001; *** = *p* < 0.001).

The mouse HDI model of HBV replication was used to assess silencing efficacy of second- and third-generation TALENs in vivo. Mice were injected with the replication-competent HBV plasmid, pCH-9/3091, TALEN-encoding plasmid, and pCI-neo FLuc, which expresses Firefly luciferase from the CMV promoter. The latter allowed for the success of HDI to be measured by bioluminescence imaging of injected mice (Figure S4). Bioluminescence imaging further confirmed that delivery was equivalent across the different groups of mice. HBsAg levels and circulating VPEs were measured by ELISA and qPCR, respectively. Analysis was carried out on serum collected from mice on days 3 and 5 post-injection. First- and second-generation TALENs targeted to the *surface* ORF resulted in greater than 90% silencing of HBsAg levels at both time points (Figure 3a). In line with data from cell culture, the third-generation Sharkey S TALEN was less efficacious and achieved 40–60% silencing over the 5-day period. Unexpectedly, all three *core*-targeting TALENs effected silencing of HBsAg, despite their target site not overlapping with the *surface* ORF. As observed in cell culture, this may be attributed to transcriptional repression by the C TALENs, which extends from the *core* target site to the S and preS1 promoter sequences. All TALENs, whether targeting the *core* or the *surface* ORF, affected suppression of circulating VPEs (Figure 3b). This is to be expected, as core and surface antigen expression is required for virion production, and as such, suppression of either will decrease circulating VPEs. Decreases in circulating VPEs ranging from 80 to 90% were observed with the second- and third-generation anti-HBV TALENs on both day 3 and day 5, with a trend of increased suppression over time. The increased inhibition is likely to represent continued action of the TALENs and accumulation of disruptive mutations. Among the second- and third-generation TALENs, the obligate heterodimeric S TALEN showed the least efficacy, and inhibition was no longer significant on day 5. Nevertheless, collectively the data showed that in vivo, the obligate heterodimeric and Sharkey TALENs function as efficiently, and in some instances more efficiently, than their first-generation counterparts. Importantly, the observed suppression occurred in the absence of any hepatotoxic effects, as serum ALT levels were not elevated in any of the mice (Figure S5).

To directly assess the effect that targeted mutagenesis has on viral gene expression, we quantified intrahepatic HBV mRNA levels by RT-qPCR. TALENs function by inducing disruptive mutations in target sequences and, unless directed against promoter or enhancer regions, are not expected to affect transcription. It was therefore unexpected that most of the first-, second-, and third-generation TALENs caused significant suppression of viral gene expression in this murine model of HBV replication (Figure 3c,d). When silencing was not significantly different from the mock-treated mice, there was nevertheless a trend towards decreased expression.

### 3.3. On- and off-Target Mutagenesis by TALENs In Vivo

**Figure 4:**
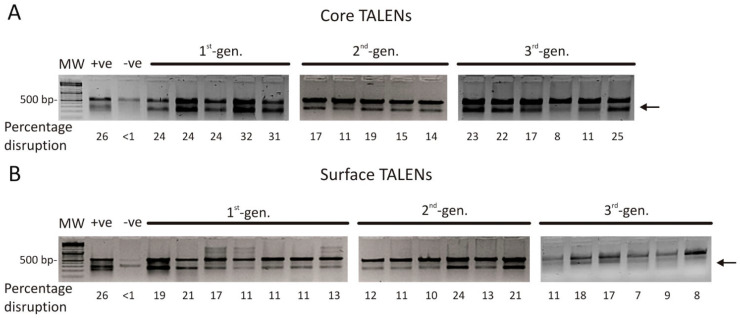
Targeted disruption of HBV-encoding DNA in murine liver samples by TALENs. To determine the levels of targeted mutagenesis mediated by the TALENs in vivo, we extracted total DNA from murine livers and *C* (**A**) and *S* ORF (**B**) target sites were amplified by PCR. PCR amplicons were subjected to CelI cleavage assay. Arrows depict on-target cleavage. MW: molecular weight marker, +ve: HBx heteroduplexes, −ve: mock.

To determine whether second- and third-generation anti-HBV TALENs induce targeted mutagenesis in viral DNA, the *core* and *surface* regions were amplified from total mouse liver DNA and subjected to the Surveyor assay. Cleavage of heteroduplexes indicated targeted mutagenesis at intended sites (Figure 4). With the exception of the obligate heterodimeric C TALEN, targeted disruption with second- and third-generation TALENs was equal to that of the first-generation TALENs (3rd-gen C vs. 1st-gen C: *p* = 0.0843; 2nd-gen S vs. 1st-gen S: *p* = 0.7455; 3rd-gen S vs. 1st-gen S: *p* = 0.0957). Interestingly, disruption observed with the obligate heterodimeric C TALEN in comparison to that of the first-generation C TALEN was lower (15.2 ± 1.51% vs. 27.0 ± 2.06%; *p* = 0.0091), but viral suppression was equivalent (Figure 2b; circulating VPEs). Furthermore, overall function of TALENs appeared to be more efficient in an in vivo setting when compared to anti-HBV action in cultured cells. Together the data demonstrate that second- and third-generation anti-HBV TALENs are at least as effective at inducing targeted disruption as their first-generation counterparts in an in vivo model of viral replication.

Because the second-generation obligate heterodimeric anti-HBV TALENs generally performed better than their third-generation Sharkey counterparts, these nucleases were chosen for further characterization. Potential off-target sites of the C and S TALENs were identified using the PROGNOS bioinformatics tool [26]. The four top hits for each TALEN were chosen, and primers described in Table S1 were used to amplify DNA from livers of mice treated with first- and second-generation C and S TALENs. The amplicons were subsequently subjected to next-generation sequencing and the reads aligned to the mouse genome to identify mutagenic events. Extensive mutations were only observed in the putative off-target site within the intron of the murine *Pah* (*phenylalanine hydroxylase*) gene. Although not statistically different, targeted disruption induced by the second-generation obligate heterodimeric C TALEN tended to be lower than that of the first-generation C TALEN (Table 1). Of note, the putative target site within the intron of the *Pah* gene is predicted to be bound by two right homodimers, and the obligate heterodimeric C TALEN is expected to target this site less efficiently. While the site within chromosome 18 (chr18:34327564-34327631) appeared to be extensively modified by both first- (81.50%) and second- (84.75%) generation TALENs, this was an artefact introduced by the co-amplification of a pseudogene with the primer set against the second C TALEN off-target site.

In addition to analyzing off-target mutagenesis, on-target disruption at the *core* and *surface* sites by first- and second-generation TALENs was also assessed. Unexpectedly, the second-generation C TALEN only caused 4.75 ± 0.75% targeted mutagenesis, whereas its first-generation counterpart induced 21.25 ± 4.17% mutagenesis in HBV DNA. This was similar to the results observed when targeted disruption was assessed using the Surveyor assay (Figure 4a). The S TALENs were more effective, inducing 45.41 ± 3.00% (first-generation S TALEN) and 32.25 ± 0.48% (obligate heterodimeric S TALEN) targeted disruption of HBV DNA in vivo. These values are much lower than the >90% reduction in plasma HBsAg levels observed in S TALEN-treated mice and may reflect an additive effect from transcriptional repression. The apparent discrepancy may also be explained by bias introduced during targeted amplification and sequencing whereby unmodified amplicons are selectively identified.

## 4. Discussion

ZFNs and TALENs are designed as left and right monomers that come together at the intended DNA target sequences, allowing their *Fok*I nuclease domains to dimerize and create a double-stranded break. However, left/left or right/right homodimers may also assemble allowing functional *Fok*I dimerization and cleavage at these unintended sites. To limit this possibility, second- and third-generation *Fok*I nuclease domains were identified that are only functional when left/right heterodimerization occurs [15,16,17]. Q486E, together with I499A and E490K, together with I538V, for example, yielded ZFN monomers that function poorly as homodimers but very efficiently as heterodimers [16]. A subsequent study identified additional modifications to the *Fok*I nuclease domain, such as ELD (Q486E, I499L, N496D) and KKR (E490K, I538K, H537R) mutations, which yielded improved obligate heterodimeric ZFNs [17]. Modifications that enhance the catalytic activity of the *Fok*I nuclease domain and improve efficacy of ZFNs have also been identified [18]. These so-called Sharkey mutations (S418P and K441E) may also reduce off-target mutagenesis of nucleases as lower dosages would be required to produce a therapeutic effect.

Here, we evaluated the use of second-generation TALENs (Q486E, I499A, E490K, and I538V obligate heterodimers) as well as third-generation TALENs (ELD and KKR obligate heterodimers) with Sharkey mutations for use against HBV. In general, the efficacy of the second-generation obligate heterodimers were on par with that of the original first-generation anti-HBV TALENs. However, although similar levels of suppression of viral replication by the second-generation obligate heterodimeric TALEN targeted to the *core* ORF were observed, lower target disruption was observed in vivo. In contrast the third-generation TALENs containing Sharkey mutations exhibited reduced silencing activity against HBV. Similar results have been reported before [28,29]. This suggests that incorporating Sharkey mutations into the TALEN architecture, and more specifically into third-generation *Fok*I nuclease domains, is deleterious to silencing activity [28,29]. The Sharkey mutations were generated by directed evolution of ZFNs and possibly provides the reason for reduced silencing activity within TALENs.

Unexpectedly, the anti-HBV TALENs reduced viral mRNA levels. The likely explanation for this observation is that, in addition to their nuclease function, the TALENs were capable of suppressing viral DNA at the transcriptional level. Although the TALENs described here do not contain transcription inhibitory domains, it has been demonstrated that ZFPs comprising only a DNA-binding domain were capable of suppressing duck hepatitis B virus (DHBV) transcription [30]. The ZFPs were targeted to the enhancer region of DHBV, which controls core and small surface protein expression, which were suppressed as a result. Unexpectedly, production of DHBV large surface protein, which is not under the control of the enhancer region, was also inhibited. Steric hindrance of RNA polymerase by the ZFPs that prevented transcription of the large surface protein gene was postulated as the mechanism for the observed indirect suppression. Another study reported inhibition of HBsAg secretion by a TALEN targeted against the *polymerase* ORF of HBV and speculated that the mechanism might be mediated by transcriptional interference [22]. Transcriptional repression by the anti-HBV TALENs may explain the results reported here. The lack of any elevation in serum ALT levels suggests the suppression is not as a result of non-specific effects.

Characterization of off-target mutagenesis in vivo using NGS suggested that second-generation obligate heterodimeric TALENs exhibit improved specificity. Extensive mutation of an intronic region of the *phenylalanine hydroxylase* gene by the first-generation C TALEN was observed, whereas the obligate heterodimeric C TALEN produced fewer mutations at this site. This observation is supported by the fact that this off-target site is predicted to be targeted by a right/right homodimer. Although lower, targeted mutagenesis induced by the obligate heterodimeric C TALEN was nevertheless substantive, suggesting that specificity of these nucleases may still require improvement.

Studies evaluating therapeutic interventions against HBV are plagued by the poor models of chronic hepatitis B. Cell culture models of viral replication, such as transient transfection of liver-derived cells or stable HBV cell lines used here, do not recapitulate all aspects of chronic infection. Cultured cells, in particular, do not model viral integration or the existence of HBV quasi-species as seen in chronic carriers. The HDI model of HBV replication used in this study, too, does not fully model the natural infection process. Of note, the mouse hepatocyte does not support cccDNA formation and as a consequence the ability of the TALENs to target this viral intermediate cannot be determined in vivo. Furthermore, HDI necessitates the co-administration of the replication-competent HBV plasmid with the TALEN-encoding plasmids, which does not model post-exposure intervention.

The ability to directly target and inactivate cccDNA makes the use of engineered nucleases, such as TALENs, a worthy avenue to be explored as a therapeutic modality for chronic HBV infection. TALEN and Cas9 function has been shown to be limited by heterochromatin [31]; more recent data suggest that TALENs fare better than the Cas9 nuclease at navigating compact DNA [32]. This is important in HBV therapy as the cccDNA has been shown to exist in a heterochromatic state [33,34]. While TALENs are obstructed by heterochromatin, activity is not completely inhibited and the nuclease is capable of navigating within heterochromatin, more so than the Cas9 nuclease, which has to separate double stranded DNA before interrogating the target site [32]. Effective targeting of the viral DNA relies on binding of the TALENs to the target sequences, which may be disrupted by escape mutations. Analysis of HBV sequencing data identified limited variability in the target sites of the TALENs described here [22]. Furthermore, targeting multiple sites within the HBV genome simultaneously will be necessary to limit viral escape. Characterization of off-target effects and development of an efficient delivery vehicle for TALEN-expressing sequences remain crucial to the eventual clinical translation of this technology. Advances in sequencing technology have yielded a wealth of information and will play an important role in identifying off-target disruption by engineered nucleases. TALENs, in particular, face the challenge of delivering two very large transgenes to the same cell to be effective. While viral vectors have been explored extensively for this purpose, the potential for recombination of repeat sequences limits their utility. Use of in vitro-transcribed mRNA with synthetic vectors may offer advantages of safety and facile large-scale manufacturing capability. TALEN technology is well-placed to fill a significant gap in anti-HBV therapeutics.

The management of HBV is plagued by poor vaccine coverage and ineffective treatment options, and as a consequence, disease burden globally, especially in resource-poor settings, remains high. Acute infections are estimated to be responsible for close to 100,000 deaths annually, but mortality from chronic hepatitis B-associated complications far exceed this number [35]. There is therefore an urgent need for curative therapies to combat chronic HBV infection. Persistence of the infection stems from the viral cccDNA, which is established as a stable episomal minichromosome during infection. Effecting a functional cure of chronic hepatitis B, involving complete suppression of cccDNA activity, is increasingly recognized as a goal capable of being achieved over that of a sterilizing cure, which necessitates the complete removal of all viral reservoirs. Engineered nucleases have the potential to directly target cccDNA and induce disruptive mutations to permanently inactivate this viral intermediate. For the eventual application of engineered nucleases in a clinical setting, undesired gene disruption at unintended target sites needs to be eliminated.

## Figures and Tables

**Table 1:** Targeted amplicon next-generation sequencing results of on-target and potential off-target sites of the anti-HBV C and S TALENs.

**TALEN**	**On-Target Site**	**Configuration**	**Percentage Disruption**	
			**1st-gen TALEN**	**2nd-gen TALEN**
Core	HBV *core* ORF	Left/Right heterodimer	21.45 ± 4.17%	4.75 ± 0.75%
Surface	HBV *surface* ORF	Left/Right heterodimer	45.41 ± 3.00%	32.25 ± 0.48%
**TALEN**	**Off-Target Site**	**Configuration**	**Percentage Disruption**	
			**1st-gen TALEN**	**2st-gen TALEN**
Core	Chromosome 8 Intergenic (*Slc10a2*) ^1^	Right/Right homodimer	0.64 ± 0.10%	0.53 ± 0.12%
	Chromosome 18 Intergenic (*Srp19*) ^1^	Right/Right homodimer	81.50 ± 1.94% <10% ^2^	84.75 ± 0.25% <10% ^2^
	Chromosome 11 Intronic (*Aspscr1*)	Right/Right homodimer	0.71 ± 0.10%	0.91 ± 0.05%
	Chromosome 10 Intronic (*Pah*)	Right/Right homodimer	12.75 ± 0.48%	8.25 ± 2.43%
Surface	Chromosome 9 Intronic (*Arih2*)	Left/Left homodimer	0.46 ± 0.11%	0.35 ± 0.08%
	Chromosome 9 Intronic (*Ppp2r3a*)	Right/Left heterodimer	0.58 ± 0.02%	0.56 ± 0.06%
	Chromosome 9 Intronic (*Stac*)	Left/Right heterodimer	0.77 ± 0.10%	0.56 ± 0.08%
	Chromosome 10 Intergenic (*Ctnna3*) ^1^	Left/Right heterodimer	0.52 ± 0.07%	0.65 ± 0.02%

^1^ Closest gene to putative target site. ^2^ Estimation of targeted cleavage *sans* pseudogene.

## Data Availability

The data presented in this study are openly available in the Sequence Read Archive (http://www.ncbi.nlm.nih.gov/bioproject/725669; reference number PRJNA725669, accessed on 28 April 2021).

## Supplementary Materials

The following are available online at https://www.mdpi.com/article/10.3390/v13071344/s1, Figure S1: Second- and third-generation FokI nuclease domain mechanism of action. Figure S2: Expression and safety profile of TALENs in cultured cells. Figure S3: HBsAg secretion in TALEN-treated HepG2.2.15 cells. Figure S4: Bioluminescence imaging of NMRI mice. Figure S5: Hepatotoxicity in TALEN-treated NMRI mice. Table S1: List of primer sequences used for amplifications of NGS library.
