# Supplementary material for: Improved Specificity and Safety of Anti-Hepatitis B Virus TALENs Using Obligate Heterodimeric FokI Nuclease Domains
Source: Viruses. 2021 Jul 12;13(7):1344. doi: 10.3390/v13071344 (PMC8310341; doi:10.3390/v13071344)
Supplement: Supplementary file 1 [file viruses-13-01344-s001.zip › viruses-1285332-supplementary.pdf]

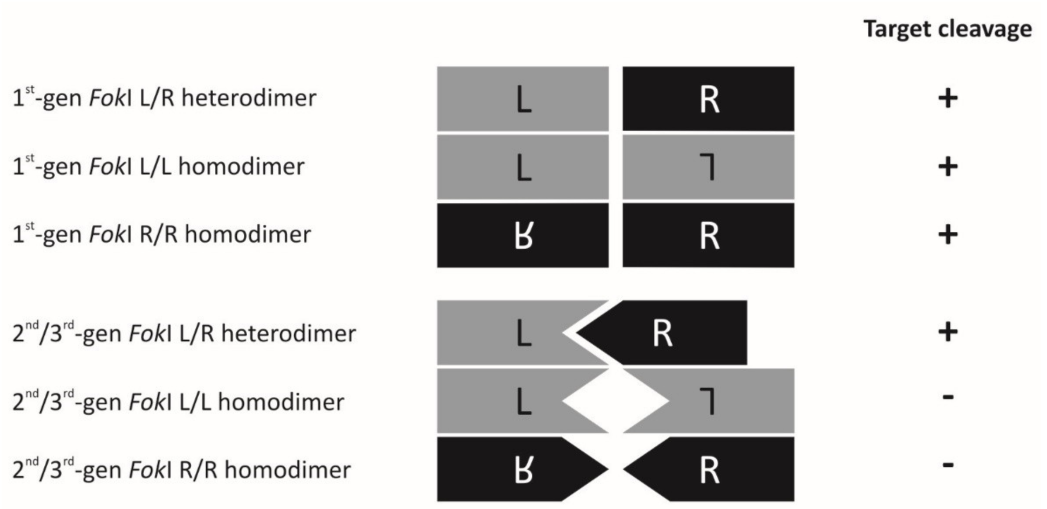

Figure S1: Second- and third-generation *Fok*I nuclease domain mechanism of action. Dimerization of first-generation nuclease domains results in cleavage regardless of monomer orientation whereas dimerization of second- or third-generation *Fok*I nuclease domains results in cleavage only when two different monomers are juxtaposed in a head-to-head orientation.

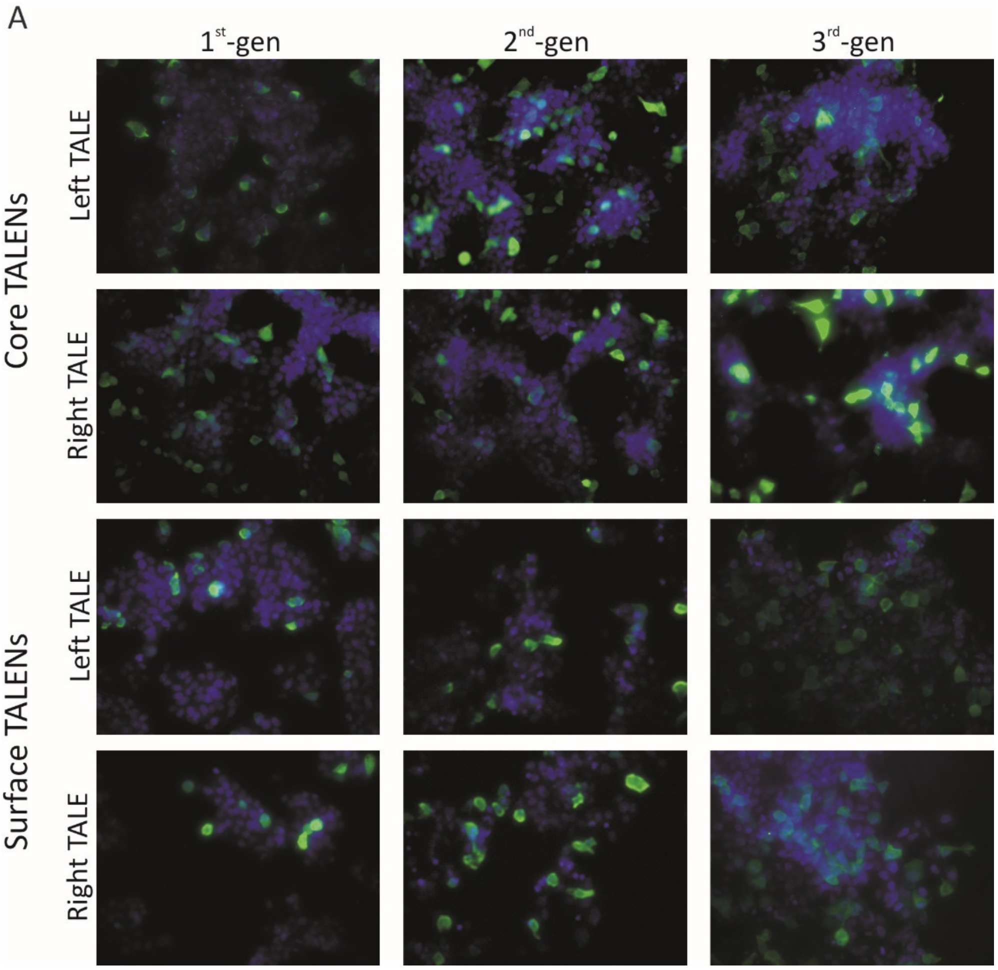

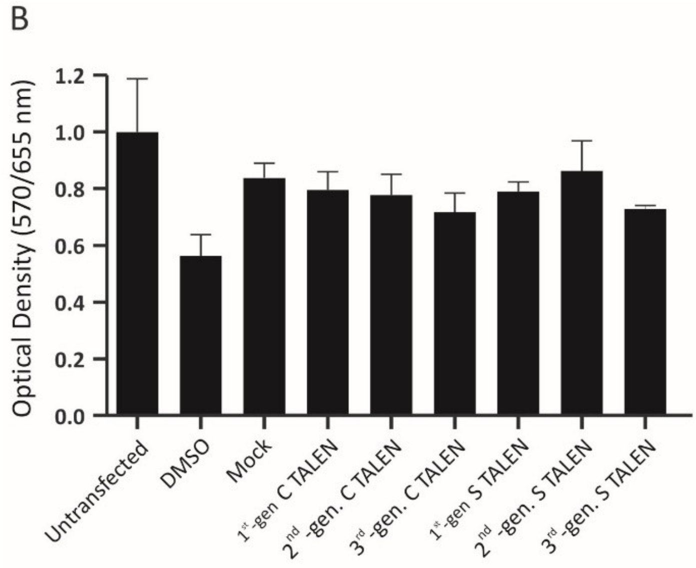

Figure S2: Expression and safety profile of TALENs in cultured cells. (a) Huh7 cells were transiently transfected with left or right TALEN monomers to determine TALEN expression in cultured cells. Immunofluorescence was detected using microscopy. The nuclei were stained with DAPI and cells were imaged at 20× magnification. (b) HEK293 cells were transiently transfected with pCH-9/3091 as well as left and right TALEN plasmids. Mock treated cells were transfected with pUC118 and pCH-9/3091 only whereas cells treated with DMSO (50%) served as the positive control. Untransfected cells did not

contain any plasmid DNA. MTT reduction was assessed 48 hours after transfection. Data obtained from this assay were averaged and normalised to untransfected controls.

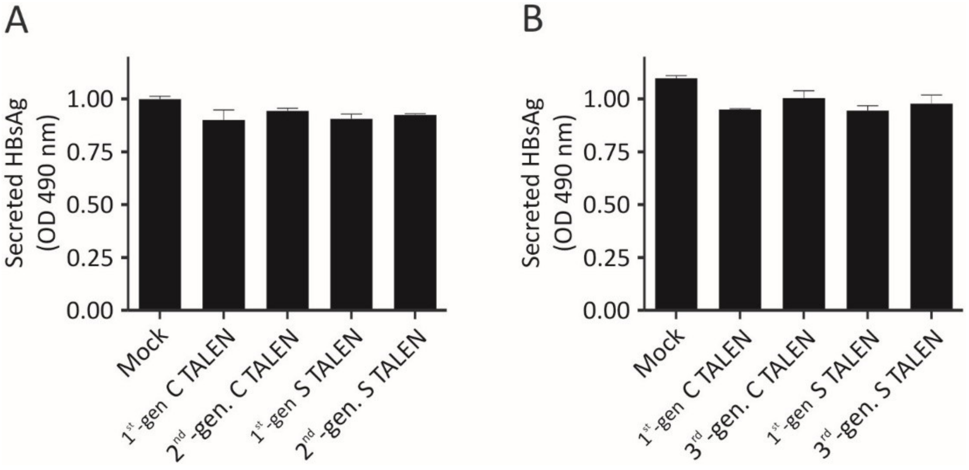

Figure S3: HBsAg secretion in TALEN-treated HepG2.2.15 cells. HepG2.2.15 cells were transfected with the indicated TALEN-expressing plasmids. After 48 hours the cells were re-seeded at 50% confluency and a day later transfected with the same TALEN plasmids. Re-seeding and transfection was carried out for a third time and 48 hours later culture supernatants were harvested and assessed for HBsAg levels.

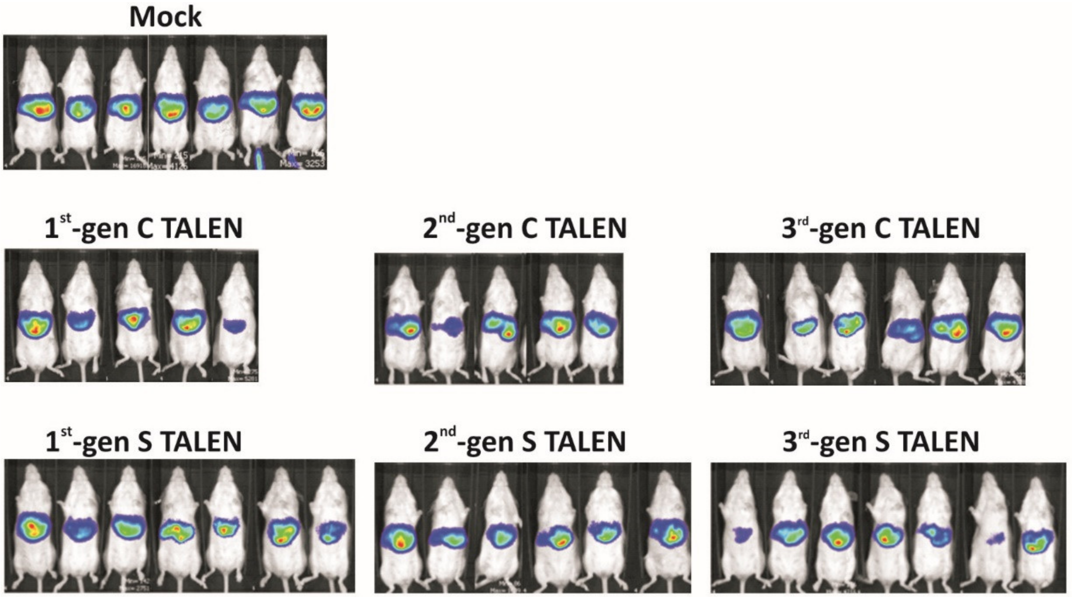

Figure S4: Bioluminescence imaging of NMRI mice. NMRI mice were hydrodynamically co-injected with pCH-9/3091, pCI-neo-FLuc and pUC118 or individual TALEN-encoding plasmids. Live imaging of Firefly luciferase expression was performed on mice on day 3 post injection.

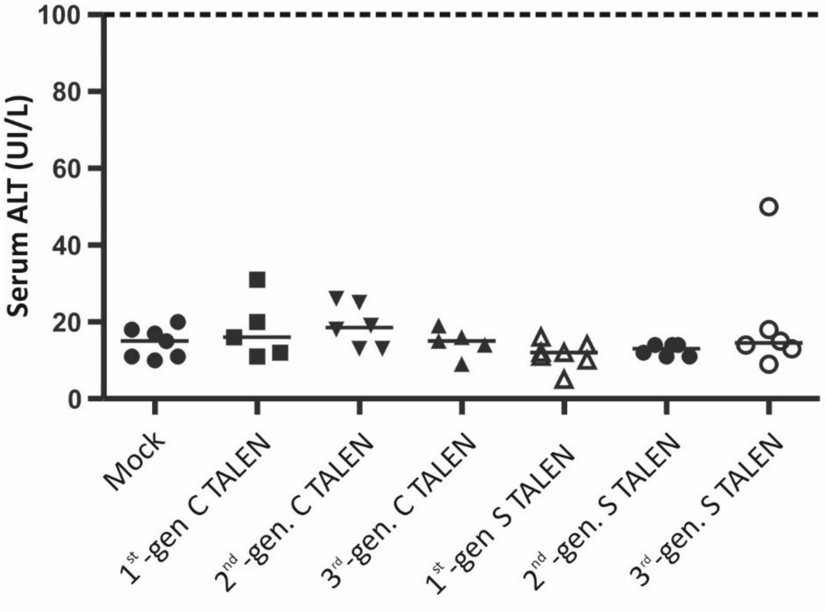

Figure S5: Hepatotoxicity in TALEN-treated NMRI mice. ALT levels were measured in serum of mock and TALEN-treated mice at day 5 after injection. No significant hepatotoxicity was observed. Data obtained from individual samples are represented where n=7 (Mock and 1st-gen S TALEN treated mice), n=6 (2nd-gen C TALEN, 2nd-gen S TALEN and 3rd-gen S TALEN treated mice) and n=5 (1st-gen C TALEN and 3rd-gen C TALEN treated mice).

Table S1: List of primer sequences used for amplifications of NGS library.

Targets	Forward primer	Reverse primer
Core Off-target 1	5’- GGTGTGTGAGAGGTGAATTCTTCC -3’	5’- TTTAGTCTGACATCTCTGGTGCCAC -3’
Core Off-target 2	5’- GATGTGGAATAAACAGTGTACTGGCAAG -3’	5’- GCAGGTCAGGAAGTAGGAGCTG -3’
Core Off-target 3	5’- GCCTGGCTGTGCTCTCTTCTTTT -3’	5’- GACAGCACAGCAGGACACAGTTG -3’
Core Off-target 4	5’- CATGGTGGGTAGGAGGAATGC -3’	5’- TCTGCATCCCTTTGAGTGGTGAC -3’
Core On target	5’- GGGCCTAAAGTTCAGGCAACTCT -3’	5’- ATTGAGATCTTCTGCGACGCGG -3’
Surface Off-target 1	5’- GGAAGCCACTGCTACTGACATG -3’	5’- CCCCCACCGAGCTGTATCCCTA -3’
Surface Off-target 2	5’- GCACAAATGGTATAGGGGCAACC -3’	5’- GATACTGACTTTGAGGGCCAGGC -3’
Surface Off-target 3	5’- CGGTTATTCCCACTTCCTGTCTCT -3’	5’- GCCCAAGGTACAAGGGTAGTTAGAATCC -3’
Surface Off-target 4	5’- GGTTTGCAGAGATTATAGAATTTCTGGGAC -3’	5’- CTGTATTAGTTACATTGGTGTTGTTGCAAC -3’
Surface On-target	5’- GGGGGAACTACCGTGTGTCTTG -3’	5’- CAGTAGTCATGCAGGTCCGGC -3’

Forward and reverse primer pairs were synthesized as four different sets with each set containing a different Multiplex identifier (MID) at their 5’-ends (Mouse #1 MID: GATCGGGCCC; Mouse #2: GATCGCGCGC; Mouse #3: GATCATCGAT; Mouse #4: GATCTGCGCA).
